# Seasonal hazards and health risks in lower-income countries: field testing a multi-disciplinary approach

**DOI:** 10.1186/1476-069X-8-S1-S16

**Published:** 2009-12-21

**Authors:** Roger Few, Iain Lake, Paul R Hunter, Pham Gia Tran, Vu Trong Thien

**Affiliations:** 1 School of International Development, University of East Anglia (UEA), UK; 2 School of Environmental Sciences, University of East Anglia (UEA), UK; 3 School of Medicine, Health Policy and Practice, University of East Anglia (UEA), UK; 4 Department of Geography, University of Social Sciences and Humanities, Viet Nam National University, Ho Chi Minh City, Vietnam; 5 Institute of Hygiene and Public Health, Ho Chi Minh City, Vietnam

## Abstract

Understanding how risks to human health change as a result of seasonal variations in environmental conditions is likely to become of increasing importance in the context of climatic change, especially in lower-income countries. A multi-disciplinary approach can be a useful tool for improving understanding, particularly in situations where existing data resources are limited but the environmental health implications of seasonal hazards may be high. This short article describes a multi-disciplinary approach combining analysis of changes in levels of environmental contamination, seasonal variations in disease incidence and a social scientific analysis of health behaviour. The methodology was field-tested in a peri-urban environment in the Mekong Delta, Vietnam, where poor households face alternate seasonal extremes in the local environment as the water level in the Delta changes from flood to dry season. Low-income households in the research sites rely on river water for domestic uses, including provision of drinking water, and it is commonly perceived that the seasonal changes alter risk from diarrhoeal diseases and other diseases associated with contamination of water. The discussion focuses on the implementation of the methodology in the field, and draws lessons from the research process that can help in refining and developing the approach for application in other locations where seasonal dynamics of disease risk may have important consequences for public health.

## Introduction

In many parts of the world, annual variations in climate produce major seasonal changes in environmental conditions. The importance of seasonality in shaping temporal patterns of disease risk and livelihood impacts, and its relative neglect by researchers, was highlighted by Chambers [1,2]. Yet seasonal dynamics still tend to have poorly specified implications for human health, especially within developing countries. Such changes are perhaps most marked on the flood plains of large rivers with high seasonal variation in discharge [3]. With the prospect of climate change bringing possible intensification of climatic seasonality in many regions [4], it is crucial to gain a better understanding of how seasonal hazards may affect health now [5]. Gold-standard epidemiological analysis of disease outcomes from hydro-meteorological hazards is challenging, partly because of the multiple transmission pathways for many water-related infections in settings of poor environmental health quality. However, there is important and undervalued practical scope for correlating data on the environmental hazard with data on health outcomes. Moreover, in order to understand the implications for public health and to design effective interventions in environmental health and health promotion, a broader reach of disciplines is also required that engages with how people living under conditions of poverty perceive and respond to such hazards [6,7].

This short paper describes the design and field testing of a multi-disciplinary approach to analysis of health risks from seasonal environmental hazards in lower-income settings, as applied to a case study from the Mekong Delta in southern Vietnam. There, the risk of gastro-intestinal and other waterborne diseases is perceived to be heightened during the annual floods, when river water spills across fields and gardens, and in many cases enters houses for a period of several weeks [8]. However, there is also contrasting anecdotal evidence that diarrhoeal disease risk from water sources may be heightened during the dry season, when river levels are lowest. Understanding of this dynamic is constrained by insufficient evidence. The paper focuses on the experimental methodology applied to this problem, its field application, and initial lessons drawn from the research.

## Methods

The methodology set out to examine the seasonal environment/health problem from hazard to outcome - combining analysis of changes in levels of environmental contamination, seasonal variations in disease incidence and a social scientific analysis of health behaviour in order to understand changing risk to public health. At the same time, recognizing the challenges for research in a developing country context, the approach places emphasis on efficiency of data collection, featuring both spatial concentration (case study methodology with intensive multidisciplinary research at ward-level) and multiple data strands to facilitate triangulation of findings. As such the methods used were simultaneously holistic but streamlined.

**Figure 1:**
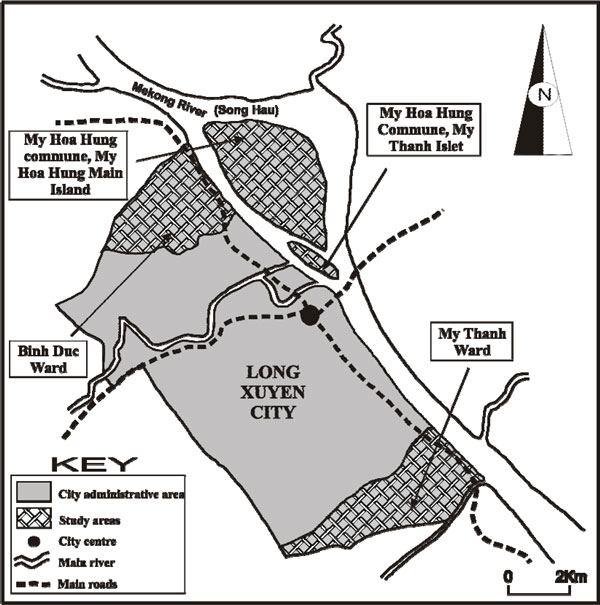
**Data collection sites within the city of Long Xuyen in the Mekong Delta, Vietnam**.

**Figure 2:**
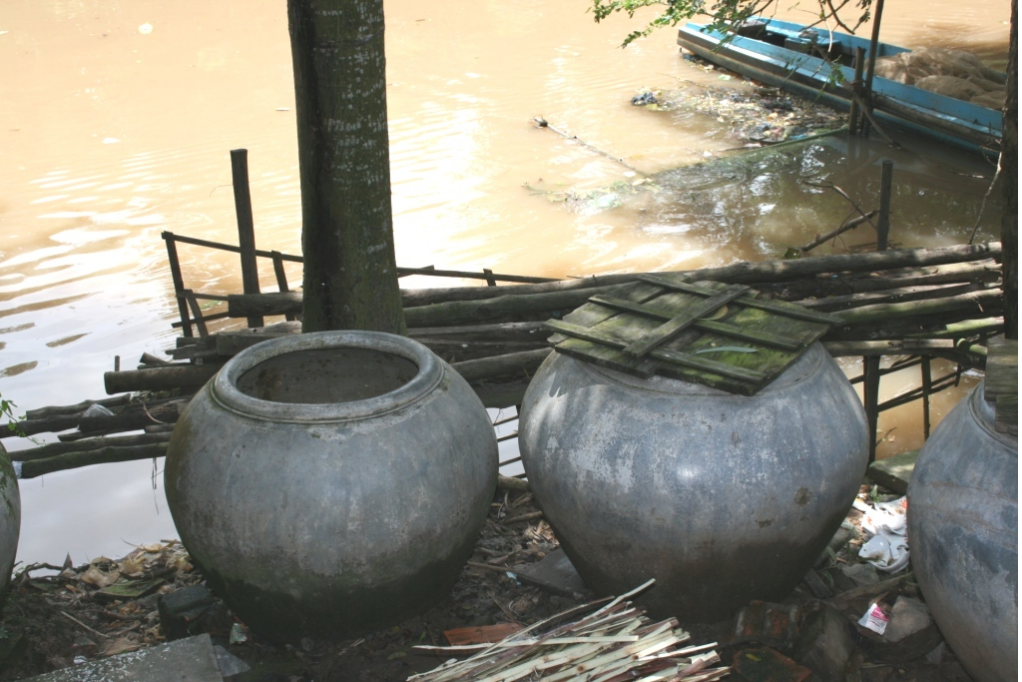
**Domestic storage pots for river water, Binh Duc, Long Xuyen**.

The research took place in four sites around Long Xuyen (population 350,000 in 2006), within the Mekong Delta in Vietnam (see Figure 1). Each year these sites face alternate seasonal extremes in the local environment due to annual flooding in the Delta. For poorer households in particular, the potential health consequences of these environmental cycles are considerable. Such households tend to rely on river water for domestic uses, including provision of drinking water (see Figure 2). Sanitation conditions are simple, with most households using simple latrines located over watercourses and fish ponds. Few have access to solid waste collection services.

**Figure 3:**
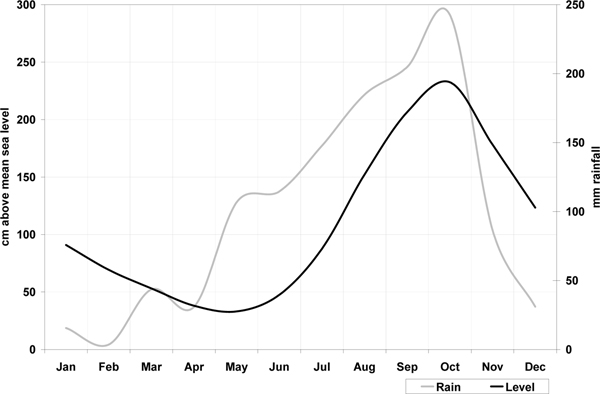
**Seasonal cycle for Long Xuyen: average monthly river water level and rainfall, 2002-2008**.

Because the key interest was the temporal dynamic of health risk, field data collection took place in four phases over a 12 months period. Fieldwork was carried out in October 2007 and 2008 during the peak of the flood season, in January 2008 in the early dry season, and in April 2008 in the late dry season. (Figure 3 indicates the monthly variations in river water levels and rainfall averaged over the period 2002-2008.) The field team worked with 120 low-income households, randomly selected from the 4 sites. These represented a population of approximately 630. The work had three main components:

### a) Water quality monitoring

Microbiological sampling was conducted to determine the potential exposure of residents to faecal contamination within the home and the immediate surroundings, and to gauge how this changes on a seasonal basis. During each of the four seasonal research phases the team tested samples from environmental water sources (including rivers, canals and ditches), together with samples of stored water from the 120 households (both drinking and non-drinking water). Each sample was analysed for Total coliform and *E. coli*, using the IDEXX Quanti-Tray^® ^system.

### b) Public health data

Four rounds of questionnaire surveys were conducted with adult representatives of the same 120 households. The survey was designed to identify the incidence of self-reported illness within the household during the previous four weeks, along with basic demographic data and information on water usage and hygiene practices at the time of each survey. Information was collected on a range of symptoms, but with follow-up questions designed especially to target reported incidences of diarrhoeal disease and skin and eye infections. Monthly data on health outcomes was also compiled from health clinic records for the study sites for the period 2002-2008. Corresponding risk data on monthly river height and rainfall data for Long Xuyen collected by the provincial hydrometeorology centre was also obtained.

### c) Household health behaviour

A questionnaire survey on health behaviour was carried out with the 120 households during the first research phase, gathering quantitative and qualitative data on perceptions of health risks and how these change during the seasons. Information on hygiene practices, specific responses to the health risks from seasonal extremes, and reasons for practices were also obtained. During the third phase, a follow-up process of 32 semi-structured interviews (8 per site) was carried out with a stratified sample of respondents to gain more in-depth, qualitative information on how perceptions, motives and constraints shape health protection behaviour. Initial findings from the first survey were used to refine and focus the more expansive discussions on key points during the interviews. Follow-up also included a series of 16 experimental 'scenario-based' interviews, using progressive storylines of seasonal dynamics to help understand perceptions of how risk changes between pre-flood and flood phases.

Analysis is ongoing and again involves multiple, inter-linked strands. It entails both interrogation within each dataset to ascertain seasonal dynamics (for example of water contamination levels, patterns of water usage, hygiene behaviour and reported disease incidence) and testing of relationships between datasets. Findings from the qualitative data are being used in the process of selecting which relationships to assess, and will bring explanatory depth to the final analysis. A joint output combining quantitative and qualitative findings will be provided to describe patterns of health impact, elucidate environmental and behavioural mechanisms of transmission, assess how responses to risk shape health outcomes, and inform prospects for successful intervention.

## Discussion

The first objective of the study was to field test a multi-disciplinary research approach. Though analysis is pending, it is possible to draw a number of lessons from progress to date in terms of research design and the processes of data collection. Here we concentrate on some key points that are likely to be applicable in many lower-income country settings.

The initial phase of fieldwork emphasized the importance of piloting a research design to fit local contexts. Household selection procedures and survey designs went through a vital iterative process of revision before and after piloting in one of the study sites. It was therefore crucial that senior team members from the different disciplines were directly involved in this stage of fieldwork. Two examples of revisions for this project included decisions to target households with children under five (to capture the perceived highest-risk group) and the decision to instruct field researchers to speak with adult women where possible (men were much less likely to express knowledge of ill-health within the household).

Another lesson learned was the recognition that, for this streamlined, integrated approach, it was appropriate to maximize sampling of high-risk population groups - both in terms of site selection and household selection. This was particularly important in order to generate sufficient data on health outcomes, bearing in mind that the research was interested in analyzing disease dynamics as opposed to community incidence *per se*. The multi-layered research approach also reduced the need to build control groups into research design. Hence all the households selected for study were on the state poverty register and in locations not protected by flood control structures. These high-risk groups also constituted the key target beneficiaries for the research, further justifying a methodological focus on the risk factors affecting them [6].

Locating, re-locating, and mapping households and sample sites can be problematic in contexts where existing maps are absent or of poor quality, houses are not numbered and there are no formal addresses. Data collection and processing could be improved through the use of GPS equipment and a standardized system of house identifiers applied across the field team from the outset of the fieldwork.

One notable value of the in-depth semi-structured interview research was that it revealed a greater complexity of household water usage than would have been derived from the questionnaire survey alone. For example each household in the survey was asked to describe a main source of drinking water currently used. However, from the qualitative interviews it became clear that members of most households drew their drinking water supply from multiple sources (including treated river water, stored rainwater, piped supplies and bottled water) at different times. This underscores the need for methodologies that can reveal a detailed understanding of multiple sources, levels of treatment and corresponding uses. The potential value of direct observation of household water use practices was raised in related research in Vietnam [9].

## Conclusion

The principal role of this exploratory project was to pilot a multi-disciplinary approach to work on seasonal health risks tailored to the research challenges and the public health priorities of lower-income countries. The research has successfully generated an integrated dataset combining information from environmental monitoring, health data and analysis of health behaviour in order to develop a multi-layered understanding of risk. The study is expected to draw valuable insights that will inform health promotion in the Mekong Delta. The researchers recognise that this is an initial study with limited scope that will affect the robustness of conclusions. However, practitioners serving poor communities in countries such as Vietnam have to make 'hard' health promotion choices based on available evidence [7], and relatively low-cost studies such as this can play an important role in guiding decisions. In a broader sense, the opportunity to test the methodology has yielded valuable lessons that will help refine the approach for application in other locations and contexts where research on seasonal environmental changes and associated disease risks has important consequences for current and future public health [10].

## Note

The peer review of this article can be found in Additional file 1.

## Authors' contributions

Please add authors' contributions section.

## Competing interests

The authors declare that they have no competing interests.

## Supplementary Material

Additional file 1 Peer review Click here for file
