# Supplementary material for: Seasonal hazards and health risks in lower-income countries: field testing a multi-disciplinary approach
Source: Environ Health. 2009 Dec 21;8(Suppl 1):S16. doi: 10.1186/1476-069X-8-S1-S16 (PMC2796494; doi:10.1186/1476-069X-8-S1-S16)
Supplement: Additional file 1 — Peer review [file 1476-069X-8-S1-S16-S1.doc]

| Article ref no. | **A1471-2105X SZ-S16** |
| --- | --- |
| Title | **Seasonal hazards and health risks in lower-income countries: field testing a multi-disciplinary approach** |
| Author(s) | Roger Few, Iain Lake, Paul R Hunter, Pham Gia Tran, Vu Trong Thien |

**Ref#1**

**General comments:**

Relevant, honest and a well written paper addressing very topical environmental health issues that will no doubt be of increasing concern in lower-income countries as a result of environmental change from urbanization coupled with impacts from greater climatic variability.

The focus on methodology is quite illuminating as this remains an often neglected discussion topic, particularly once research findings emerge which then attracts more attention over methodological issues and lessons.

**Major compulsory revisions:**

PAGE 3 Introduction

In the introduction the authors fail to acknowledge the seminal work of Robert Chambers on seasonal biases and seasonality. This emission is quite notable when considering the similarity in methods and the multi-disciplinary approach, of course Chambers’ work focused on rural areas, and this (Few et al) study applies the multi-disciplinary seasonality approach to the urban context nevertheless referencing the early work of Chambers would bring some conceptual depth, for example see:

Chambers, R. (1983) Rural Development: Putting the Last First. Longman, Harlow.

Chambers, R. (1993) Challenging the Professions: Frontiers for Rural Development. Intermediate Technology, London. (See Chapter 4 Health, Agriculture, and Rural Poverty: Why Seasons Matter).

**Minor essential revisions:**

PAGE 4 Last Paragraph

Include figure for total population of Long Xuyen.

PAGE 5 First Paragraph

When is the peak(s) of the dry season: January or April, or January and April? Graph would be useful to illustrate this see comment in Discretionary revisions.

FIGURES

Map in Figure 1 is important for spatial representation but could be enhanced by following points:

- Include boundaries of the sample sites particularly in Binh Duc Ward and My Thanh Ward where there is no natural features to delineate the Wards from the rest of the city.
- In the map key identify what the different shades are (2x grays and white island).
- Include north direction and map scale.

**Discretionary revisions:**

PAGE 4 Last Paragraph

Include population density of Long Xuyen.

PAGE 5 First Paragraph

Graph(s) illustrating Mekong water level and rainfall levels over 12-month period would be useful.

PAGE 6 *c) Household health behaviour*

During follow-up phase did you use seasonal calendars / timelines / resource mapping, if not may be useful to comment on these kinds of data collection tools that could have been designed and applied for this research context, where you facing time constraints during these interviews?

PAGE 8 Last Paragraph

“One notable value of the qualitative research was that it revealed a greater complexity of household water usage than would have been derived from the questionnaire survey alone”.

Better to be more precise here to avoid potential ambiguity as some may argue questionnaire surveys can also be qualitative, for example:

One notable value of the in-depth semi-structured interviews was that it revealed a greater complexity of household water usage than would have been derived from the questionnaire survey alone.

Chart illustrating

FIGURES

Figure 2, photo of sampling, does not add particular value to the article, a general photo of human settlements on the Mekong or of community members abstracting drinking water from the Mekong may be more suitable to illustrate the hazardous conditions.

**Ref#2**

**General comments:**

The emphasis in this paper is on the methodology and gives valuable insights into the problems associated with this type of project. The paper is interesting and identifies issues concerning implementation that are useful. However, there is a need for data to be included if the paper is to be acceptable for publication.

**Minor essential revisions:**

*Abstract* – peri-urban not peri-ban

# Responses to Reviewers’ comments

**Ref#1**

**Major compulsory revisions:**

In the introduction, reference has been added to the work of Chambers in highlighting the importance of seasonality in shaping temporal patterns of disease risk and livelihood impacts, and its relative neglect by researchers. We have not gone into greater depth on this, however, because this work mainly refers to what Chambers terms the ‘dry season bias’ of urban-based researchers (ie in favouring work in rural areas only in dry seasons) – which is not so directly relevant to the subject of this paper.

**Minor essential revisions:**

A figure for the total population of Long Xuyen has been added.

The timing of the dry season and the associated fieldwork has been clarified in the text, and a new graph (figure 3) has been provided showing average monthly water levels/rainfall.

The map (figure 1) has been re-drawn, and now addresses all the requested revisions.

**Discretionary revisions:**

It has not been possible to source a reliable population density figure for the city.

The requested graph has been provided (see above).

In the follow-up phase we did indeed use a more participatory data collection tool – this involved working through ‘scenario-based’ storylines of seasonal dynamics; information on this tool has been added to the text.

The ambiguity concerning regarding qualitative methods and questionnaires surveys has been addressed as requested.

A photograph showing river water storage vessels alongside a river channel has been added to illustrate the hazard.

**Ref#2**

**General comments:**

As the first Reviewer notes and endorses, this short paper focuses on methodology and lessons that can be derived from it, and it is important to keep that focus of the paper distinct. Data and findings will be the focus of additional papers.

**Minor essential revisions:**

Correction has been made to the spelling of ‘peri-urban’.
